# Frondanol, a Nutraceutical Extract from *Cucumaria frondosa*, Attenuates Colonic Inflammation in a DSS-Induced Colitis Model in Mice

**DOI:** 10.3390/md16050148

**Published:** 2018-04-30

**Authors:** Sandeep B. Subramanya, Sanjana Chandran, Saeeda Almarzooqi, Vishnu Raj, Aisha Salem Al Zahmi, Radeya Ahmed Al Katheeri, Samira Ali Al Zadjali, Peter D. Collin, Thomas E. Adrian

**Affiliations:** 1 Department of Physiology, College of Medicine and Health Sciences, United Arab Emirates University, P.O. Box–17666, Al Ain, UAE; sanjanachandran25@gmail.com (S.C.); rajvishnu@uaeu.ac.ae (V.R.); 201400107@uaeu.ac.ae (A.S.A.Z.); 201304934@uaeu.ac.ae (R.A.A.K.); 201304302@uaeu.ac.ae (S.A.A.Z.); 2 Department of Pathology, College of Medicine and Health Sciences, United Arab Emirates University, P.O. Box–17666, Al Ain, UAE; saeeda.almarzooqi@uaeu.ac.ae; 3 Coastside Bio Resources, Deer Isle, ME 04627, USA; pcollin48@gmail.com

**Keywords:** Frondanol, *Cucumaria frondosa*, DSS colitis, colon inflammation

## Abstract

Frondanol is a nutraceutical lipid extract of the intestine of the edible Atlantic sea cucumber, *Cucumaria frondosa,* with potent anti-inflammatory effects. In the current study, we investigated Frondanol as a putative anti-inflammatory compound in an experimental model of colonic inflammation. C57BL/6J male black mice (C57BL/6J) were given 3% dextran sodium sulfate (DSS) in drinking water for 7 days to induce colitis. The colitis group received oral Frondanol (100 mg/kg body weight/per day by gavage) and were compared with a control group and the DSS group. Disease activity index (DAI) and colon histology were scored for macroscopic and microscopic changes. Colonic tissue length, myeloperoxidase (MPO) concentration, neutrophil and macrophage marker mRNA, pro-inflammatory cytokine proteins, and their respective mRNAs were measured using ELISA and real-time RT-PCR. The tissue content of leukotriene B4 (LTB4) was also measured using ELISA. Frondanol significantly decreased the DAI and reduced the inflammation-associated changes in colon length as well as macroscopic and microscopic architecture of the colon. Changes in tissue MPO concentrations, neutrophil and macrophage mRNA expression (F4/80 and MIP-2), and pro-inflammatory cytokine content (IL-1β, IL-6 and TNF-α) both at the protein and mRNA level were significantly reduced by Frondanol. The increase in content of the pro-inflammatory mediator leukotriene B4 (LTB4) induced by DSS was also significantly inhibited by Frondanol. It was thus found that Frondanol supplementation attenuates colon inflammation through its potent anti-inflammatory activity.

## 1. Introduction

Inflammatory bowel disease (IBD) refers to Crohn’s disease (CD) or ulcerative colitis (UC), which involve both small and large intestines. IBD is characterized by chronic inflammation with mucosal ulceration in the intestinal tract [1]. The prevalence of IBD is increasing (150–250/100,000) in developed nations [2], and greatly diminishes quality of life because of the morbidity associated with it such as pain, vomiting, and diarrhea. IBD also increases the risk of colorectal cancer [2]. The current mainstream therapies for IBD are sulfasalazine, corticosteroids, immunosuppressive agents such as azatriopine, and anti-tumor necrosis factor-α antibodies, either as single agents or in combination, to inhibit aberrant immune response and inflammation. However, the adverse effects associated with these treatments over prolonged periods as well as the concomitant high relapse rate of the disease limits their use [3]. With the lack of effective treatment, in addition to the associated side effects and costs, many IBD patients turn to complementary and alternate therapy [4].

The marine environment has been of great interest for drug discovery in the last several decades, as researchers acknowledge its promising potential for new drug leads. To date, only a few drugs from marine sources have been isolated, developed, and approved to treat diseases such as cancer, but the search continues [5]. Sea cucumbers have been used for hundreds of years as food and folk medicine in the communities of Asia and the Middle East [6]. Sea cucumbers are also referred to as ‘marine ginseng’, and they contain numerous useful specific compounds that could have broad applications in agricultural, nutraceutical, pharmaceutical, and cosmeceutical products [7]. Frondanol is a US-patented nutraceutical lipid extract of the intestines of the edible Atlantic sea cucumber *Cucumaria frondosa.* Frondanol has potent anti-inflammatory activity. It has been purported to suppress inflammation in the adjuvant arthritis rat model and ear edema mice model when it is administered either orally or applied topically [8]. Frondanol exhibits potent inhibitory activity on both 5-lipoxygenase (5-LOX) and 12-lipoxygenase (12-LOX) pathways, suppressing the production of 12-hydroxyeicosatetraenoic acid (12-HETE), 5-hydroxyeicosatetraenoic acid (5-HETE), and leukotriene B4 (LTB4) in human polymorphonuclear cells [8]. There is considerable evidence for the involvement of the 5-LOX pathway in colitis. The products of 5-LOX, 5-HETE, and LTB4 are markedly increased in the dextran sodium sulfate (DSS) model [9,10,11]. When DSS-treated animals are given a 5-LOX inhibitor or an LTB4 antagonist, colonic shortening as well as histological and inflammatory scores were improved in the mouse model of colonic inflammation [9,11,12,13]. Therefore, we hypothesized that Frondanol may attenuate the inflammatory responses seen in IBD. Various experimental animal models of IBD have been developed to mimic the pathophysiological processes that characterize UC [14,15]. Perhaps the most accepted model of colon inflammation is the murine model of colitis that utilizes oral administration of DSS, a murine chemical colitogen to induce UC [16]. In the present study, we investigated the efficacy of Frondanol in attenuating the colonic inflammation induced by DSS in mice.

## 2. Results

### 2.1. Effect of Frondanol on Disease Activity Index (DAI) and Colon Length

**Figure 1:**
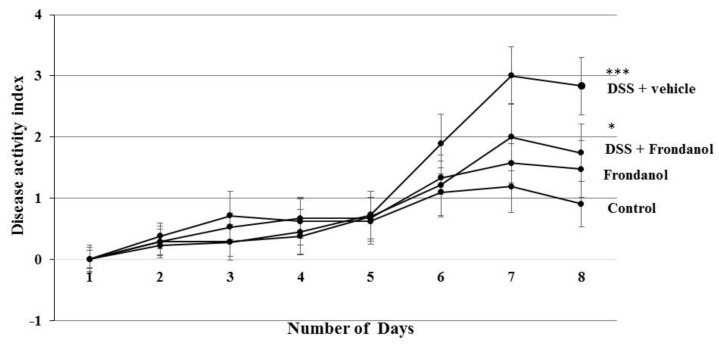
Effect of Frondanol on disease activity index (DAI) and colon length. Dextran sodium sulfate (DSS) treatment significantly increased the DAI score. Frondanol treatment significantly decreased the DAI score compared to the DSS-treated group. Data were obtained from *n =* 8 animals in each group and are expressed as means ± SEM (control vs. DSS, *** *p* < 0.001 and DSS vs. Frondanol + DSS. * *p* < 0.05, was obtained by one-way ANOVA followed by Tukey’s multiple comparison test).

**Figure 2:**
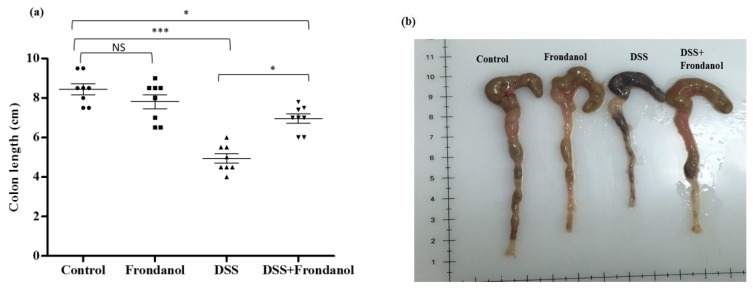
Effect of Frondanol on colon length. The average colon length (cm) (**a**,**b**) was significantly decreased in the DSS-treated group. Frondanol treatment significantly prevented the shortening of colon length compared to the DSS-treated group. Data were obtained from *n =* 8 animals in each group and are expressed as means ± SEM (control vs. DSS *** *p* < 0.001, DSS vs. DSS + Frondanol, and control vs. DSS + Frondanol * *p* < 0.05. NS indicate not significant was obtained by one-way ANOVA followed by Tukey’s multiple comparison test).

In C57BL/6J mice, 3% DSS in drinking water induced distinct features of ulcerative colitis. There was a marked and significant increase in the DAI score in the DSS-treated group (*p* < 0.001). Frondanol administration significantly prevented the increase in the DAI score in DSS-treated animals (*p* < 0.05, Figure 1). However, Frondanol alone had no significant effect on the DAI score compared with the control. DSS treatment significantly reduced colon length (cm) when compared to the control (*p* < 0.001). Frondanol treatment in of the DSS group significantly prevented the shortening of colon length (*p* < 0.05, Figure 2a,b). However, Frondanol alone did not affect colon length compared to the control.

### 2.2. Effect of Frondanol on Microscopic Architecture

**Figure 3:**
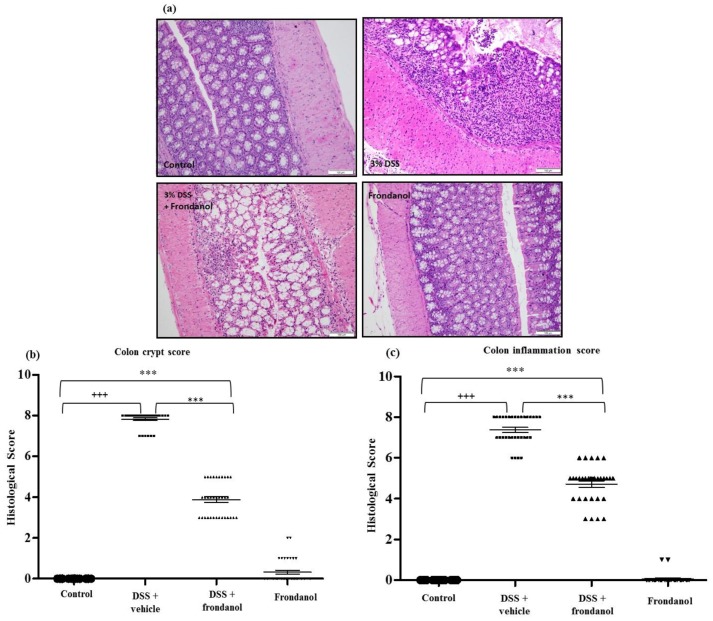
Effect of Frondanol on colon histology. (**a**) Hematoxylin and Eosin (H&E) staining was carried out to provide material for microscopic scoring (Scale Bars: 100 μm). Microscopic analysis shows typical architecture of the colon with normal thickness of the submucosa, muscle layer, and regular crypt structure in the mucosa in the control. The DSS-treated colon section shows focal loss of crypts and surface epithelium with inflammation reaching up to the submucosa. Frondanol protected the microscopic architectures in the DSS-treated group. Crypt distortion score and colon inflammation score (**b**,**c**) were significantly higher in the DSS-treated group. Frondanol treatment significantly lowered both crypt distortion and colon inflammation scores. All of the histology pictures are represented at 20× magnification. Data were obtained from *n =* 8 animals in each group and are expressed as means ± SEM. (control vs. DSS +++ *p* < 0.0001, DSS vs. DSS + Frondanol, and control vs. DSS + Frondanol *** *p* < 001, obtained by one-way ANOVA followed by Tukey’s multiple comparison test).

The healthy normal control colon section depicted typical architecture of a colon with normal thickness of the submucosa and muscle layer, as well as regular crypt structure in the mucosa. In the DSS-treated control group, colonic inflammation reached up into the submucosa with focal loss of crypts and surface epithelium. In contrast, the Frondanol-treated DSS group had intact epithelium with minimal loss of crypt and inflammation compared the normal control group (Figure 3a).

The most reproducible histological abnormality in the DSS-treated control group was excessive crypt damage with edema, collapse, or complete destruction (Figure 3a). Crypt damage score was significantly worse in DSS-mice compared with untreated controls and Frondanol-treated DSS animals (*p* < 0.0001 and *p* < 0.001, respectively, Figure 3a,b). However, the crypt damage score was still significantly higher in the Frondanol-treated DSS group compared with healthy controls (*p* < 0.001). Frondanol alone had no effect on the crypt damage score. The colonic inflammation score was markedly and significantly increased in the DSS-treated mice compared to untreated control animals (*p* < 0.0001, Figure 3c). Frondanol treatment in the DSS-treated group significantly prevented the increase in colonic inflammation score compared to DSS treatment alone (*p* < 0.001, Figure 3c). The inflammation score was still significantly elevated in the Frondanol-treated DSS group compared to healthy controls, indicating only partial, but significant protection of both colonic crypt damage and inflammation by Frondanol (*p* < 0.001, Figure 3c). Frondanol alone (without DSS) had no significant effect on the colon inflammation score compared to controls. These results confirm that Frondanol has a protective effect on the colonic micro architecture, damaged by DSS treatment.

### 2.3. Effect of Frondanol on the Expression of Myeloperoxidase (MPO) Protein, F4/80 Macrophage Marker mRNA, and Macrophage Inflammatory Protein-2 (MIP-2) mRNA Concentrations

**Figure 4:**
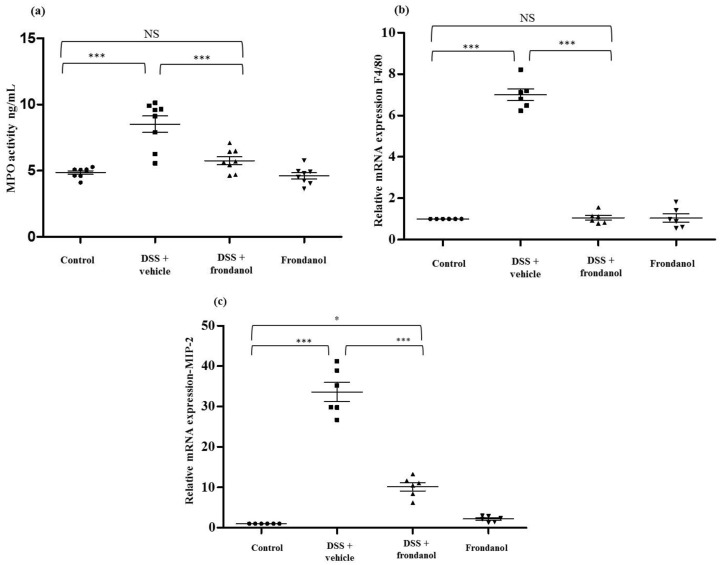
Effect of Frondanol on MPO concentration, F4/80 neutrophil markers, and MIP-2 mRNA expression. DSS treatment significantly enhanced neutrophil infiltration, as marked by an increase in (**a**) MPO concentration and (**b**,**c**) F4/80 and MPI-2 mRNA expression compared to the control. Frondanol administration significantly decreased (**a**) MPO activity, F4/80, and MIP-2 mRNA expression in the DSS-treated group. Frondanol alone did not affect (**a**) MPO concentration, (**b**) F4/80, or (**c**) MIP-2 mRNA expression. MPO concentrations were quantitated using ELISA. F4/80 and MIP-2 mRNA expression studies were carried out using real-time RT-PCR. Data were obtained from *n =* 8 animals for MPO concentration and *n =* 6 animals for mRNA expression studies in each group, and are expressed as means ± SEM (control vs. DSS, and DSS vs. DSS + Frondanol, *** *p* < 0.001; control vs. DSS + Frondanol, * *p* < 0.05. NS indicate not significant was obtained by one-way ANOVA followed by Tukey’s multiple comparison test).

Myeloperoxidase (MPO) is an enzyme secreted from activated neutrophils and is commonly used a marker to assess the level of neutrophil infiltration at a site of inflammation. MPO concentrations were measured in colon homogenates using an ELISA assay. MPO concentrations were markedly and significantly increased in the DSS group compared to the control (*p* < 0.001, Figure 4a). Frondanol administration significantly prevented the DSS-induced increase in MPO concentrations (*p* < 0.01), indicating the role of Frondanol in mitigating inflammatory response induced by DSS. Frondanol alone did not significantly affect MPO concentrations (Figure 4a). In DSS-induced colitis, both neutrophil and macrophage infiltration was significantly increased. We measured mRNA expression of F4/80 protein as a marker of macrophage infiltration. F4/80 mRNA was significantly increased in the DSS-treated group compared to the control (*p* < 0.001). Frondanol treatment in the DSS-treated animals significantly (*p* < 0.01) prevented F4/80 mRNA expression (*p* < 0.001, Figure 4b). However, Frondanol alone had no effect on F4/80 mRNA expression. MIP-2 mRNA expression massively and significantly increased in the DSS-treated group compared to the control (*p* < 0.001, Figure 4c). Frondanol administration in the DSS-treated group significantly prevented the increase in MIP-2 mRNA expression (*p* < 0.001, Figure 4c). However, the group receiving Frondanol treatment following DSS still had an MIP-2 mRNA level higher than that of the control (*p* < 0.05, Figure 4c). Frondanol alone had no effect on MIP-2 mRNA expression. These results confirm that Frondanol limits the infiltration of macrophages and neutrophils due to damage induced by DSS.

### 2.4. Effect of Frondanol on Pro-Inflammatory Cytokine Protein and mRNA Concentrations

**Figure 5:**
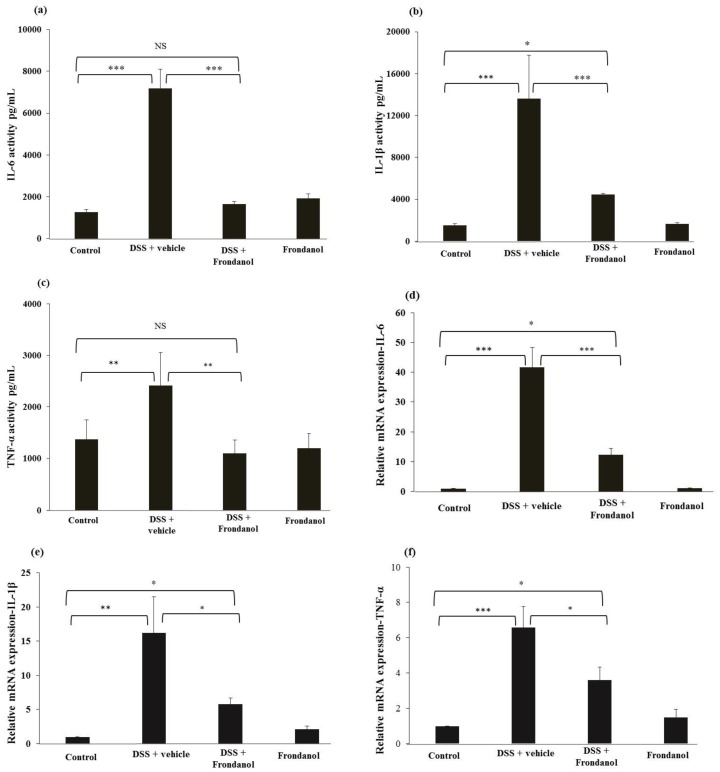
Effect of Frondanol on pro-inflammatory cytokine protein and mRNA expression. Frondanol significantly inhibits pro-inflammatory cytokines both at the protein level (IL-6 (**a**), IL-1β (**b**) and the TNF-α (**c**)) and mRNA expression level in the DSS-treated group (Il-6 (**d**), IL-1β (**e**), TNF-α (**f**)). Pro-inflammatory cytokine proteins were measured using ELISA and the expression of their respective mRNAs were measured in using real-time RT-PCR. Data were obtained from *n =* 8 animals for ELISA and *n =* 6 animals for mRNA expression studies in each group, and are expressed as means ± SEM. (ELISA results of IL-6 and IL-1β: control vs. DSS, and DSS vs. DSS + Frondanol, *** *p* < 0.001; control vs. DSS + Frondanol, * *p* < 0.05. ELISA of TNF-α: control vs. DSS, and control vs. DSS + Frondanol, ** *p* < 0.001. mRNA expression of IL-6: control vs. DSS, and DSS vs. DSS + Frondanol, *** *p* < 0.001. IL-1β: control vs. DSS ** *p* < 0.01; DSS vs. DSS + Frondanol, and control vs. DSS + Frondanol, * *p* < 0.05. TNF-α: control vs. DSS *** *p* < 0.001; DSS vs. DSS + Frondanol, and control vs. DSS + Frondanol, * *p* < 0.05. NS indicate not significant was obtained by one-way ANOVA followed by Tukey’s multiple comparison test).

DSS treatment caused massive increases in protein concentrations of the pro-inflammatory cytokines interleukin-1β (IL-1β), interleukin-6 (IL-6) (*p* < 0.001, Figure 5a,b), and tumor necrosis factor-α (TNF-α), (*p* < 0.01, Figure 5c), as well as the relative expression of the mRNAs for these respective cytokines (IL-6 and IL-1β, *p* < 0.001; TNF-α, *p* < 0.01, Figure 5d–f). Frondanol administration prevented increases in pro-inflammatory cytokines and their respective mRNA expression in response to DSS treatment (*p* < 0.001 for IL-6, IL-1β and *p* < 0.01 for TNF-α cytokines. *p* < 0.001 for IL-6, and *p* < 0.05 Il-1β and TNF-α mRNAs). In control animals, Frondanol alone had no effect on the expression of the cytokines or their respective mRNAs. These results indicate that Frondanol strongly inhibits the pro-inflammatory cytokine response induced by DSS.

### 2.5. Effect of Frondanol on Leukotriene B4 (LTB4)

**Figure 6:**
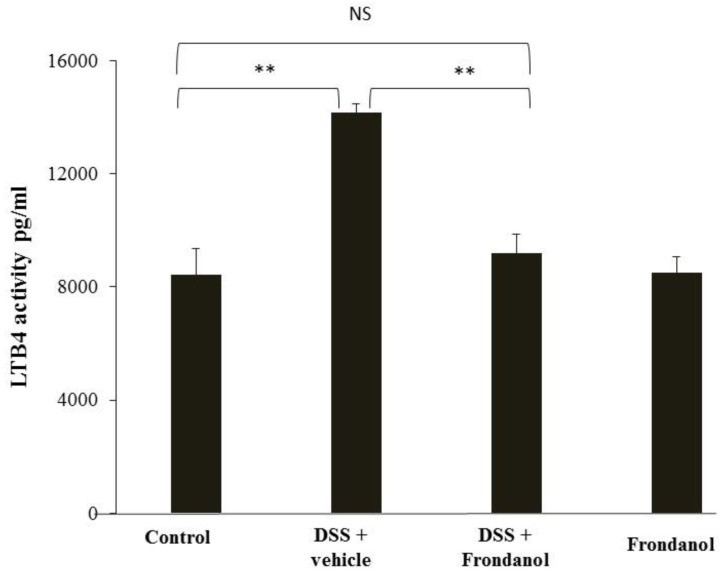
Effect of Frondanol on LTB4 concentrations. Frondanol administration significantly reduced LTB4 level in the DSS-treated group. Data were obtained from *n =* 8 animals in each group and are expressed as means ± SEM (control vs. DSS and DSS vs. DSS + Frondanol, ** *p* < 0.01. NS indicate not significant was obtained by one-way ANOVA followed by Tukey’s multiple comparison test).

LTB4 is secreted from neutrophils, and a markedly increased response is seen in inflammatory conditions. Frondanol is reported to inhibit the function of 5-lipoxygensase, which is upstream of leukotriene B4 synthase [8]. In our DSS model, LTB4 concentrations were measured using a commercially available ELISA kit. DSS administration markedly increased LTB4 concentrations compared with the control (*p* < 0.01). Frondanol treatment completely prevented this increase (*p* < 0.001) in DSS-treated animals, reflecting its inhibitory effect on LTB4 production. As expected, Frondanol alone had no effect on the LTB4 level in control animals (Figure 6).

## 3. Discussion

In the current study, we investigated the effects of Frondanol on DSS-induced experimental colitis. The DSS-induced colitis model is a widely accepted model of colon inflammation for both understanding the disease process as well as screening the efficacy of various anti-inflammatory natural and dietary compounds because of its clinical relevancy to human ulcerative colitis [17].

Frondanol is a US-patented nutraceutical non-polar extract of the intestines of the edible Atlantic sea cucumber, *Cucumaria frondosa,* which has potent anti-inflammatory properties. This extract should not be confused with Frondanol A5, which is a polar extract of skin from the same animal that has been shown to have potent anti-cancer properties [18]. Frondanol suppresses inflammation in the adjuvant-induced arthritis model [18], developed in rats as a model for screening compounds that might prove useful in the treatment of rheumatoid arthritis in humans. In this model, Frondanol (10 mg/kg by gavage) was reported to be at least as effective as phenylbutazone (10 mg/kg) and more effective than hydrocortisone (10 mg/kg) [8]. In the mouse ear inflammatory edema model using croton oil (containing phorbol esters and arachidonic acid) Frondanol was applied topically and suppressed the edematous response by 84% [8]. Frondanol was also reported to reduce the proliferation of peripheral blood mononuclear cells stimulated by the mitogen concanavalin A. Frondanol at 0.1% suppressed concanavalin A-induced proliferation by 85% in human and 95% in sheep mononuclear cells [19]. In the mixed lymphocyte reaction (MLR), Frondanol reduced thymidine incorporation by 40–52% at 0.025% and by 82–98% at 0.05%; this was similar to the inhibition caused by the cyclosporin A control, which reduced the MLR by 37–75% at 100 ng/mL and by 81–90% at 500 ng/mL [8,19]. Cell viability was maintained by these concentrations of Frondanol, indicating the lack of toxicity of the compound.

With regard to the mechanism of action of the effects of Frondanol, the extract has reported potent inhibitory activity on 5-LOX, 12-LOX, and cyclooxygenase pathways. Frondanol suppressed the production of 5-HETE and LTB4 from human polymorphonuclear cells by 53% and 62%, respectively, at a concentration of 0.01% [8]. Similarly, Frondanol suppressed the production of 12-HETE by 44% at the same concentration [8]. Frondanol also caused a concentration-dependent reduction of PGE2 production. In separate studies on the effect of Frondanol on 5-LOX activity, dose-responsive effects were seen. Frondanol at 0.01% inhibited the production of 5-HETE and LTB4 by 84.7 ± 0.8% and 75.2 ± 7.2%, respectively. At 0.1% Frondanol inhibited the production of 5-HETE and LTB4 by 98.4 ± 0.8% and 91.9 ± 0.6%, respectively (unpublished results). While Frondanol suppresses the production of pro-inflammatory eicosanoids, it increases the activity of the anti-inflammatory 15-LOX, as evidenced by a concentration-dependent increase in the production of 15-HETE (Personal Communication, Peiying Yang, MD Anderson Cancer Center, Houston, TX, USA).

The inhibitory effects of Frondanol on 5-LOX and 12-LOX activity could be explained, at least in part, by some of the known constituent fatty acids in the extract. For example, 12-methyl tetradecanoic (12-MTA) acid is a major constituent of Frondanol (20–30% by weight) and this branched chain fatty acid has potent 5-LOX and 12-LOX inhibitory activity [20]. Topical administration of 12-MTA has been reported to reduce MPO activity by inhibiting neutrophil infiltration at the site of injury when the cornea was damaged by chemical injury [21]. Another fatty acid constituent of Frondanol is myristoleic acid, which also potently inhibits 5-LOX activity [22]. Myristoleic acid is found at lower concentrations in Frondanol, but has potent 5-LOX inhibitory effects. These fatty acids are likely to contribute to the anti-inflammatory effects of Frondanol. Astaxanthin is a carotenoid present in Frondanol that has marked anti-inflammatory effects, and has also been shown to suppress inflammation in the DSS colitis model [23].

In the present study, Frondanol markedly suppressed the inflammation driven by oral DSS administration. This was evidenced by marked decreases in the disease activity index (DAI), reduction in colon length, and macroscopic and microscopic architecture changes that reflected the inflammation. Frondanol also attenuated the increases in MPO concentrations, neutrophil and macrophage mRNA expression (F4/80 and MIP-2), and pro-inflammatory cytokine production (IL-1β, IL-6 and TNF-α) at both at the protein and mRNA levels, as well as the increase in the expression of the pro-inflammatory mediator LTB4.

Prevention of the DSS-induced increase in MPO concentrations indicates that Frondanol prevents the accumulation of polymorphonuclear granulocytes in DSS-induced colitis tissue, since MPO activity reflects the increase in neutrophil accumulation in the submucosa due to the severity of colitis [24]. The increased levels of LTB4 in colonic tissue from DSS-treated mice are strongly implicated in attracting both neutrophils and macrophages to the site of injury, thus amplifying the inflammatory cascade in IBD [10].

Along with neutrophils, the infiltration of macrophages also increases during the course of colitis, indicating its involvement in the inflammatory process [25,26]. To determine the level of macrophage infiltration, we quantitated the F4/80 mRNA expression level. F4/80 is considered a surrogate biomarker for infiltrating macrophages, suggesting the role of innate immune cells in DSS-induced colitis [27]. Frondanol significantly decreased F4/80 mRNA expression, thus suggesting its role in preventing the further recruitment of macrophages in colitis. MIP-2 is a chemoattractant secreted by both neutrophils and macrophages, and plays a central role in recruiting an increased number of neutrophils and macrophages at the site of injury [28]. Furthermore, in the DSS-induced model of colitis, MIP-2 plays an integral role in the recruitment of neutrophils and macrophages [28]. Additionally, it has also been shown that overexpression of MIP-2 enhances the severity of DSS-induced colitis, Ohtsuka et al. [29] suggesting its crucial role in the development of DSS-induced colitis. Thus, we quantitated the expression of MIP-2 mRNA by real time PCR. The MIP-2 mRNA level was significantly upregulated in DSS-induced mice, and Frondanol beneficially and significantly decreased the MIP-2 mRNA expression level. These studies suggest that Frondanol prevents the upregulation of MIP-2, thereby decreasing neutrophil accumulation as reflected by MPO concentration. These data reflecting macrophage statuses in the treated animals could partially explain Frondanol’s anti-inflammatory role in protecting against DSS-mediated colitis. 

Frondanol significantly suppressed the DSS-induced production of pro-inflammatory cytokines (IL-6, IL-1β, and TNF-α) both at the protein and mRNA levels. When Frondanol was administered by oral gavage (10 mg/kg weight) in the adjuvant arthritis model, it was as effective as phenylbutazone and much more effective than hydrocortisone at a similar dose [8]. In another model of inflammation, mouse ear edema was induced by croton oil, which contains phorbol esters and arachidonic acid (potent mediators of inflammation), and was subsequently reduced by 84% when Frondanol was applied topically [8].

There is considerable evidence for the involvement of the 5-LOX pathway and COX-2 in the DSS model of colitis [9,11,12,13,30,31]. A recent study reported the measurement of lipid inflammatory mediators throughout the different phases of inflammation [9]. In the induction phase, prostaglandin E2 (PGE2) and thromboxane A2 (TBA2) increased 2-fold [9]. In the acute phase, the production of n-6 fatty acid-derived leukotrienes increased by more than 10-fold, while that of the anti-inflammatory n-3 fatty acid-derived leukotrienes decreased [9]. In the recovery phase, the production of protectin D1 increased 3-fold [9]. The 5-LOX inhibitor B-98 reduced the colonic shortening and histological inflammatory score in DSS-treated mice [11]. In the DSS model, a peroxisome proliferator-activated receptor gamma (PPARγ) agonist, 5-ASA (that blocks nuclear factor kappa B-induced production of pro-inflammatory eicosanoids), a LOX inhibitor (AA-861), or an LTB4 receptor antagonist all prevented erosions in the large bowel [12]. The anti-inflammatory agent 5-ASA and the LTB4 receptor antagonist also prevented the shortening of the colon [12]. The COX inhibitor indomethacin and the thromboxane A2 inhibitor OKY-046 were ineffective [12]. This study indicates the importance of lipoxygenase products in this model [12]. Intracolonic administration of a selective 5-LOX inhibitor, zileuton, accelerated healing in a rat model of colitis [32]. In a DSS study using a LOX inhibitor (AA-861) and linolenic acid supplementation, the combination was able to suppress the excessive chloride secretion that results in diarrhea. Linolenic acid is metabolized to the anti-inflammatory eicosanoids PGE1 and TBA1 and prevents the formation of leukotrienes, while AA-861 is a selective 5-LOX and LTB4 inhibitor [13]. In another study, DSS caused a marked inflammatory response in the colon and a subsequent 5-fold increase in eicosanoid production [31]. This increase was completely prevented by treatment with olsalazine (a prodrug that delivers 5-ASA directly into the colon) [31]. In a study investigating the production of eicosanoids in patients with relapsing ulcerative colitis, luminal PGE2 and LTB4 concentrations were positively correlated with disease activity and were reduced to near normal levels by successful treatment with specific inhibitors of the prostaglandin and leukotriene pathways [33]. Indeed, as surrogate biomarkers, prostaglandin and leukotriene expressions appear to be better indicators of treatment outcomes for relapsing ulcerative colitis than the traditional clinical indices of disease activity [33].

In addition to its inflammatory role, the 5-LOX pathway also appears to be involved in colitis-associated neoplasia. 5-lipoxygenase is overexpressed in all adenomatous colonic polyps and in colonic carcinomas, but it is not expressed in normal mucosal cells or hyperplastic polyps [34]. Indeed, 5-LOX has been proposed as a marker for early pancreatic intraepithelial neoplastic lesions [35]. Lipoxygenase inhibitors block the growth of human colonic cancer cells in culture and induce apoptosis in these cells [34]. Other proteins in the 5-LOX pathway are also expressed in colonic cancer cells, including LTB4 receptors [36]. Furthermore, the LTB4 receptor antagonist etalocib blocks cancer cell growth both in vitro and in vivo and enhances the effect of gemcitabine on colon cancer growth in a mouse xenograft model [36]. Finally, the reported observation that Fondanol activates 15-LOX is interesting since, when overexpressed, this enzyme suppresses colitis-associated colon cancer through inhibition of the IL-6/STAT3 signaling pathway [37,38].

## 4. Materials and Methods

### 4.1. Chemicals and Reagents

Dextran Sulfate Sodium (DSS), molecular weight 36,000–50,000 Da, was purchased from Sigma-Aldrich (St. Louis, MO, USA). Frondanol was supplied by Coastside Bio Resources (Deer Isle, ME, USA). Enzyme-linked immunosorbent assay (ELISA) kits for the measurement of concentrations of myeloperoxidase (MPO), interleukin-6 (IL-6), interleukin-1β (IL-1β), tumor necrosis factor-α (TNF-α), and leukotriene B4 (LTB4) in colon tissues were obtained from R&D systems (Minneapolis, MN, USA). RNA extraction was performed using RNeasy kits obtained from Qiagen (Hilden, Germany). SYBR green and reverse transcription kit to convert mRNA into cDNA was purchased from Applied Biosystems (Foster City, CA, USA). Primers for the real time (RT-PCR) quantification of mRNA expression were supplied by Macrogen Inc. (Seoul, Korea).

### 4.2. Experimental Animals

C57BL/6J male mice (10–12 weeks old) were used for the study. Mice were housed individually in controlled environmental conditions at a room temperature 23 ± 2 °C, with a 12-h light-dark cycle and ad libitum access to food and water. Animal experiments were performed in accordance with protocols approved by the UAEU Animal Care and Research Ethical Committee on 6 June 2017 (ERA_2017_5567).

### 4.3. Experimental Design, Induction of Colitis and Tissue Collection

The mice were randomly assigned to four groups. Group 1: control untreated, Group 2: control treated with Frondanol, Group 3: DSS-induced colitis untreated, and Group 4: DSS-induced colitis treated with Frondanol. Fresh 3% DSS solutions were made every morning in autoclaved drinking water. The two DSS groups of mice were given 3% DSS for 8 days (day 0–day 7) as previously described, while the control groups received only autoclaved tap water [24]. The two treated groups were given Frondanol (100 mg/kg body weight/per day) using refined sunflower oil as a vehicle by gavage, for a total volume of 150 µL. The control groups were administered only 150 µL of refined sunflower oil. Mice were euthanized on the eighth day by intraperitoneal (IP) injection of pentobarbital (80 mg/kg body weight). After laparotomy, the colon was excised, and the length of each colon was measured carefully and photographed to determine colonic shortening induced by DSS treatment. Subsequently, each colon was flushed thoroughly with ice-cold normal saline (0.9% NaCl) several times to remove remnant DSS and then the colon was cut into several circular pieces. For histology, a small circular piece of tissue (5 mm) was taken, measuring 5 cm from distal to caecum, from all animals and fixed in 4% buffered formalin for histology (hematoxylin and eosin staining). Remaining pieces of tissue were snap-frozen in liquid nitrogen and then stored at −80 °C for subsequent extraction for ELISA and mRNA expression studies.

### 4.4. Macroscopic Assessment or Disease Activity Index (DAI)

To establish disease activity index (DAI), body weight, stool consistency, and rectal bleeding were monitored in each mouse daily to score the severity of colitis using a previously described scoring system (Table 1) [39]. The sum of the three values constitutes the DAI, resulting in a total clinical score ranging from a minimum of 0 to a maximum of 12.

### 4.5. ELISA

Concentrations of MPO and cytokines (IL-6, IL-1β, and TNF-α) and LTB4 were quantified using ELISA assays, according to the manufacturer’s protocol. Briefly, approximately 100 mg pieces of colon tissue were weighed and homogenized using a T-25 digital Ultra TURRAX homogenizer (Staufen, Germany) at 14,000 RPM in 1 ml ice-cold phosphate-buffered saline solution (PBS, pH = 7.2) containing a proteases cocktail (St. Louis, MO, USA) at 4 °C. The homogenate was centrifuged at 12,000× *g* for 10 min. The supernatant was used for the measurement of MPO, IL-6, IL-1β, TNF-α, and LTB4. The results were calculated as pg/mg tissue.

### 4.6. Colonic Cytokine mRNA Content Determined by Real-Time PCR

Total RNA was extracted from frozen pieces of colon using the RNeasy Mini-Kit. The quality and the quantity of total RNA were assessed using a NanoDrop 2000 spectrophotometer (Thermo Fisher Scientific Inc., Waltham, MA, USA). Reverse transcription was carried out using a high capacity cDNA Reverse Transcription kit. Real-time polymerase chain reaction (PCR) was performed using the QuantStudio 7 Flex Real-Time PCR System (Applied Biosytems, Culver City, CA, USA) with SYBR Select Master Mix (Applied Biosystems). The obtained data was normalized using 18s RNA as a reference gene and the 2^−ΔΔ*CT*^ method was used as a relative quantification method for mRNA expression [40]. Primer sequences for each mRNA are shown in Table 2 [41,42].

### 4.7. Histological Scoring

After overnight fixation in 4% buffered formalin, tissues were placed in 100% ethanol overnight. Tissues were then embedded in paraffin for routine histology. Four transverse sections (2 µm) taken from each colonic sample were stained with hematoxylin and eosin (H&E) and examined by light microscopy (Zeiss, Stuttgart, Germany). Colonic crypt distortion and inflammation were evaluated microscopically. The histological scoring was performed by an experienced pathologist (SAM) in a blinded fashion. Histological examination of tissues from DSS-treated animals involved a scoring system that includes the evaluation of the severe disruption of tissue architecture, the severity of edema, the massive immune cell infiltration indicating inflammation, crypt damage, and any significant area of complete epithelial denudation. Colonic crypt distortion and inflammation were scored out of 8. The histology scoring system is indicated in Table 3 and Table 4 [39]. All the histology pictures are represented at 20× magnification.

### 4.8. Statistics

All statistical analysis was carried out using SPSS Software (Armonk, NY, USA). Comparisons between groups were performed by one-way analysis of variance (ANOVA), followed by Tukey’s post-hoc test for multiple comparisons. Data are plotted as means ± SEM in the figures. *p* values < 0.05 were considered statistically significant.

## 5. Conclusions

These studies demonstrate that Frondanol, a non-polar extract from the edible Atlantic sea cucumber *Cucumaria frondosa*, has marked anti-inflammatory properties in a mouse model of colitis. These anti-inflammatory effects are most likely attributable to the inhibitory effects of the extract on 5-LOX, 12-LOX, and cyclooxygenase enzymes, with perhaps a contribution from activation of the 15-LOX pathway. This nutraceutical may be of value in the treatment of this condition. Furthermore, since both the LOX and COX pathways were implicated in the development of precancerous colonic polyps, the use of such broad-spectrum LOX and COX inhibitors together with the activation of 15-LOX may be valuable in the chemoprevention of colitis-associated colon cancer.

## Figures and Tables

**Table 1:** Disease activity index score.

Weight Loss	Score	Stool Consistency	Score	Rectal Bleeding	Score
No loss	0	Normal	0	No Blood	0
1–5%	1	Loose stool	2	Heme occult + ve and visual pellet bleeding	2
5–10%	2	Diarrhea	4	Gross bleeding and blood around anus	4
10–20%	3				
>20%	4				

**Table 2:** Real time PCR primer sequence.

Name	Forward	Reverse
IL-6	5′-ACAACCACGGCCTTCCCTACTT-3′	5′-CACGATTTCCCAGAGAACATGTG-3′
IL-1β	5′-ACCTGCTGGTGTGTGACGTT-3′	5′-TCGTTGCTTGGTTCTCCTTG-3′
TNF-α	5′-CACGTCCGTAGCAAACCACCAA-3′	5′-GTTGGTTGTCTTTGAGATCCAT-3′
F4/80	5′-TGTGTCGTGCTGTTCAGAACC-3′	5′-AGGAATCCCGCAATGATGG-3′
MIP-2	5′-GGATGGCTTTCATGGAAGGAG-3′	5′-TTGCTAAGCAAGGCACTGTGC-3′
18S	5′-AAATCAGTTATGGTTCCTTTGGTC-3′	5′-GCTCTAGAATTACCACAGTTATCCAA-3′

**Table 3:** Crypt score = product of percentage of crypt change and crypt distortion, graded out of a maximum score of 8.

Crypt Grade		Quantification of the Percentage of Crypt Change		Crypt Distortion—Graded Based on the Extent of Involvement	
Grade 0:	Intact crypt	1	1–25%	0	No crypt distortion
Grade 1:	Shortening and loss of basal 1/3 of crypts	2	26–50%	1	1–25%
Grade 2:	Loss of basal 2/3 of crypts	3	51–75%	2	26–50%
Grade 3:	Loss of entire crypt with intact surface epithelium	4	76–100%	3	51–75%
Grade 4:	Loss of both entire crypt and surface epithelium (erosion)			4	76–100%

**Table 4:** Score of inflammation = grade of inflammation × percentage of involvement, graded out of a maximum score of 8.

Inflammation Graded	Percentage of Inflammation Involvement of Mucosal Surface Area		Hyperplastic Epithelium—Graded Based on the Extent of Involvement	
0	0	No inflammation	0	None
1	1	1–25%	1	1–25%
2	2	26–50%	2	26–50%
3	3	51–75%	3	51–75%
4	4	76–100%	4	76–100%
